# Home-based intervention for non-Hispanic black families finds no significant difference in infant size or growth: results from the Mothers & Others randomized controlled trial

**DOI:** 10.1186/s12887-020-02273-9

**Published:** 2020-08-18

**Authors:** Heather M. Wasser, Amanda L. Thompson, Chirayath M. Suchindran, Barbara D. Goldman, Eric A. Hodges, Meredith J. Heinig, Margaret E. Bentley

**Affiliations:** 1 Department of Nutrition, University of North Carolina, 135 Dauer Drive, CB# 7400, Chapel Hill, NC 27599-7400 USA; 2 University of North Carolina, Chapel Hill, NC USA; 3 University of California, Davis, CA USA

**Keywords:** Infant feeding, Sedentary behavior, Sleep, Obesity prevention

## Abstract

**Background:**

Non-Hispanic black (NHB) infants are twice as likely as non-Hispanic white infants to experience rapid weight gain in the first 6 months, yet few trials have targeted this population. The current study tests the efficacy of “Mothers & Others,” a home-based intervention for NHB women and their study partners versus an attention-control, on infant size and growth between birth and 15 months.

**Methods:**

Mothers & Others was a two-group randomized controlled trial conducted between November 2013 and December 2017 with enrollment at 28-weeks pregnancy and follow-up at 3-, 6-, 9-, 12-, and 15-months postpartum. Eligible women self-identified as NHB, English-speaking, and 18–39 years. The obesity prevention group (OPG) received anticipatory guidance (AG) on responsive feeding and care practices and identified a study partner, who was encouraged to attend home visits. The injury prevention group (IPG) received AG on child safety and IPG partners only completed study assessments. The primary delivery channel for both groups was six home visits by a peer educator (PE). The planned primary outcome was mean weight-for-length z-score. Given significant differences between groups in length-for-age z-scores, infant weight-for-age z-score (WAZ) was used in the current study. A linear mixed model, using an Intent-To-Treat (ITT) data set, tested differences in WAZ trajectories between the two treatment groups. A non-ITT mixed model tested for differences by dose received.

**Results:**

Approximately 1575 women were screened for eligibility and 430 were enrolled. Women were 25.7 ± 5.3 years, mostly single (72.3%), and receiving Medicaid (74.4%). OPG infants demonstrated lower WAZ than IPG infants at all time points, but differences were not statistically significant (WAZ_diff_ = − 0.07, 95% CI − 0.40 to 0.25, *p* = 0.659). In non-ITT models, infants in the upper end of the WAZ distribution at birth demonstrated incremental reductions in WAZ for each home visit completed, but the overall test of the interaction was not significant (*F*_2,170_ = 1.41, *p* = 0.25).

**Conclusions:**

Despite rich preliminary data and a strong conceptual model, Mothers & Others did not produce significant differences in infant growth. Results suggest a positive impact of peer support in both groups.

**Trial registration:**

ClinicalTrials.gov, NCT01938118, 09/10/2013.

## Background

There has been an approximate 60% increase in overweight among infants and toddlers in the past few decades [1, 2], a concern given associations between large infant size and rapid postnatal growth with subsequent child and adult overweight [3, 4] and future co-morbidities [5, 6]. Behavioral determinants associated with large infant size and rapid growth include short durations of exclusive breastfeeding (EBF) or any breastfeeding (BF) [7], introduction of complementary foods (CF) before 4 months [8, 9], short sleep duration [10, 11], early emergence of potentially obesogenic diets [12–15], and high levels of screen-time [16–18]. Importantly, there is growing evidence on modifiable factors associated with early life feeding and care behaviors, including infant feeding attitudes, intentions, self-efficacy, and social support [19–24], as well as parental feeding styles [25, 26] and appropriate interpretation of infant fussiness [27–29].

One priority population for intervention is non-Hispanic black (NHB) infants. As compared to non-Hispanic white (NHW) infants, NHB infants have a higher prevalence of obesity [2], are twice as likely to experience rapid weight gain in the first 6 months [30], and are less likely to be breastfed [31]. Studies among low-income, NHB mothers have documented a prevalent feeding pattern of formula, solids, and juice in the first 3 months [28, 32, 33], and NHB infants are more likely than NHW infants to have a daily sleep duration of < 12 h, to have a TV in the bedroom, and to consume sugar-sweetened beverages and fast food [30].

The purpose of the current study is to report the effectiveness of “Mothers & Others,” a home-based, responsive feeding and care intervention delivered by trained peer educators (PEs) to NHB pregnant women and their study partners. The intervention began during the second trimester of pregnancy and continued through 12 months postpartum, with final follow-up at 15 months. To increase social support for the targeted behaviors, intervention women identified a study partner at baseline, who was encouraged to attend all home visits and was provided their own set of intervention materials. The attention-control group received a similar number of contacts focused on injury prevention and identified a study partner, who only completed study assessments. Specifically, we compare the effect of the intervention versus the attention-control on the main outcome of infant size and growth between birth and 15 months postpartum.

## Methods

### Overall study design

Mothers & Others was a two-group randomized controlled trial (RCT) conducted between November 2013 and December 2017 among 430 NHB pregnant women and families living in central North Carolina. The study began when women were at 28 weeks gestation (baseline) and had a final assessment when infants were 15 months old, with interim assessments at birth, 1, 3, 6, 9, and 12 months of infant age. The primary delivery channel was home visits by PEs. Institutional review board approval has been granted by the University of North Carolina, Office of Human Research Ethics.

### Participants and recruitment

Pregnant NHB women were recruited by trained recruitment specialists in prenatal clinics serving two hospitals in central North Carolina. Eligible women were English-speaking, 18–39 years, < 28 weeks’ gestation, expecting a singleton pregnancy, planning to stay in the area, and willing to identify a study partner, an “other.” At baseline, mothers identified a study partner by answering the question, “Who is the person, other than a doctor or healthcare professional, that is most important to your decision-making about infant care or that will be involved in caring for the infant during the first few months after his/her birth?” Labor and delivery (L&D) exclusion criteria were multiple birth, premature birth (< 36 weeks), mother or infant having a hospital stay > 7 days after delivery, birthweight < 2500 g, or diagnosis of a condition significantly affecting feeding or growth.

### Interventions

Participants in the obesity prevention group (OPG) received eight home visits, an information toolkit, and four newsletters designed to provide anticipatory guidance (AG) and support for enactment of six targeted infant feeding and care behaviors: breastfeeding (EBF until 6 months, continued BF until 12 months); adoption of a responsive feeding style; use of non-food soothing techniques for infant crying; appropriate timing and quality of CF; minimization of TV/media; and, promotion of age-appropriate infant sleep. Six home visits were delivered by a PE at 30- and 34-weeks gestation and 3-, 6-, 9-, and 12-months postpartum. PEs were AA women who were required to have a MS/MPH degree in a health-related field or a BS/BA degree in a health-related field plus two or more years of experience providing individual or group counseling. Additionally, the PE for the intervention group had breastfed her own children and received over 100 h of training in breastfeeding, CF, and infant behavior during the first 6 months of study preparation. Intervention families could receive up to two additional home visits by an International Board Certified Lactation Consultant (LC) after hospital discharge, at any time of their choosing. The AG curriculum, described previously in detail [32], was informed by multiple expert resources, including the Baby Behavior program [33], Ages & Stages Learning Activities [34], the Start Healthy Feeding Guidelines [35], and the American Academy of Pediatrics Nutrition Handbook [36].

Content for the injury prevention group (IPG) was based on the injury prevention AG published in AAP Bright Futures [37]. An attention-control design was chosen to control for differences in social support provided by the PE, while providing content unrelated to the targeted health behaviors. Women received the same number of home visits, a toolkit, and newsletters. The PE for the control group had previous experience in the supervision of young children and received over 100 h of training during the study preparation phase in the prevention of Sudden Infant Death Syndrome, proper installation of infant car safety seats, and household injury prevention measures. While women in both groups identified a study partner, IPG partners only completed study assessments; they were not encouraged to attend home visits or given their own set of study materials. An overview of the content for each treatment group is provided as supplemental material (Supplemental Table 1).

### Randomization and data collection

Due to the influence of hospital practices on breastfeeding outcomes [38], randomization was stratified by hospital—each prenatal clinic served one large, metropolitan hospital—using a computer-generated sequence, block size of 50, and 1:1 allocation ratio. The project director, who had no direct contact with participants, was responsible for generating the random number table and uploading it to REDCap [39], a secure, online database maintained by the North Carolina Center for Translational and Clinical Sciences Institute. After completing the baseline assessment, the PE randomized the participant using the randomization functionality in REDCap. Blinding was not maintained for PEs and participants after treatment allocation, as participants were made aware of the intervention groups during the consent process and PEs delivered the differing intervention content.

### Measures

#### Background characteristics

Maternal, study partner, and household demographic information was collected at baseline. Participants self-identified their race/ethnicity using categories in the National Health and Nutrition Examination Survey (NHANES). For labor and delivery information, research assistants in the prenatal clinics monitored hospital deliveries and promptly notified study staff, who administered a brief survey to mothers, inclusive of infant sex, gestational age, birthweight, and presence of L&D exclusion criteria. Depression was measured using the Center for Epidemiologic Studies – Depression (CES-D) scale, with presence of depressive symptoms defined as a score of 16 or higher [40, 41].

#### Outcome

The primary outcome listed in the protocol and trial registration was lower weight-for-length z-score (WLZ) at 15 months, smaller change in WLZ between 0 and 15 months, and lower likelihood of overweight (WLZ ≥ 95th percentile) at 15 months. All z-scores, including weight-for-age (WAZ) and length-for-age (LAZ), were calculated using the World Health Organization 2006 international growth standards [42]. Infant birth weight and length were self-reported by mothers during a telephone-based survey, which was administered shortly after birth to assess continued eligibility. Anthropometrics at subsequent time points were directly measured by the one PE assigned to the respective treatment group. Each PE had prior experience in research-related anthropometric measurement and received approximately 16 h of additional training at study start in guidelines used in NHANES [43]. Infant weight was measured on a digital scale (Tanita BD-585 Digital Baby Scale) to the nearest 10 g. Recumbent length was measured to the nearest 0.1 cm using a portable length board (O’Leary Length Board). All anthropometrics were done in triplicate and their mean was used in analysis. Despite PEs being trained and achieving strong inter-rater reliability, there were systematic differences in LAZ between treatment groups, with OPG infants significantly shorter at multiple time points. The relative technical error of measurement (TEM) for LAZ was 0.12 and 0.08% for the OPG PE and IPG PE, respectively. The relative TEM remained high from the initial 3-month visit (OPG = 0.13% and IPG = 0.05%) to the final 15-month visit (OPG = 0.10% and IPG = 0.10%). Given the difference in LAZ, the analysis of the primary outcome proceeded with WAZ, including the mean difference in WAZ between treatment groups and proportion overweight, defined as WAZ ≥2 SD of the WHO 2006 international growth standards [40]. Results for WLZ are also presented for the reader.

### Sample size and Power calculation

The target sample size was 468 families based on power analyses showing a minimum of 354 mother-infant pairs (177 per group) would allow detection of an effect size of ≥0.30 in infant WLZ at 15 months. This was based on an estimated mean WLZ of 0.34 ± 1.04 SD from our preliminary observational cohort study [16, 25, 28, 44]. To achieve the minimum sample size of 354 infants at study end, we incorporated a 12% loss of mothers due to L&D criteria and 20% due to attrition.

### Statistical analysis

Descriptive statistics by visit and treatment group were run for all variables. Differences between treatment groups in baseline characteristics and completers and non-completers of home visits were tested using chi-square for dichotomous and ANOVA for continuous variables. All primary analyses were conducted on an Intent-to-Treat (ITT) basis. Randomized subjects who experienced any L&D exclusion criteria were not included in the analyses.

To test the impact of the intervention on infant WAZ, we used a series of cross-sectional, multivariable, linear regression models. For each model, WAZ was entered as the dependent variable and treatment indicator as the independent variable. A linear mixed effects model was used to test whether the weight trajectories differed between the two treatment groups. The longitudinal WAZ score between groups at birth, 3-, 6-, 9-, 12- and 15-months was the dependent variable and treatment indicator, visit time, and treatment-by-time interaction were independent variables. To examine the effects of the intervention on the tails of the WAZ distribution, a three-level categorical variable was created based on initial weight status at birth (“lower birth WAZ” if birth WAZ was at or below − 1 SD, “middle birth WAZ” if birth WAZ was between − 1 SD and + 1 SD, and “upper birth WAZ” if birth WAZ was at or above + 1 SD). A multivariable, linear regression model was run with change in WAZ between birth to 15 months as the dependent variable and treatment, categorical size at birth (lower, middle, or upper WAZ), and treatment-by-size interaction as the independent variables. To examine the effects of the intervention on WAZ by dose received, all models were repeated as non-ITT, in which dose was entered as a continuous independent variable ranging from 0 to 6, based on the number of educational home visits the participant completed. Finally, models also tested for differences by type of study partner.

All models were first run unadjusted, followed by an adjusted model, which included breastfeeding status as a covariate and any variables found significantly different between treatment groups at baseline or between completers and non-completers of home visits. Finally, multiple imputation using multivariate normal regression was used to account for missing data in each of the multivariable, cross-sectional models and the linear regression model examining change in WAZ between birth to 15 months; the linear mixed effects model accounts for missingness, rendering imputation unnecessary. While missing observations can lead to less precision, including larger standard errors and less power, as well as biased parameter estimates, there were no differences in the parameter estimates and levels of significance between our imputation and complete-case models; thus only the results of the complete-case analysis are shown. Further, we were unable to identify any auxiliary variables with a correlation > 0.4, adding strength to the assumption that the data are missing at random (MAR). Stata v15 was used for all analyses. Significance was set at *P* < .05.

## Results

**Fig. 1:**
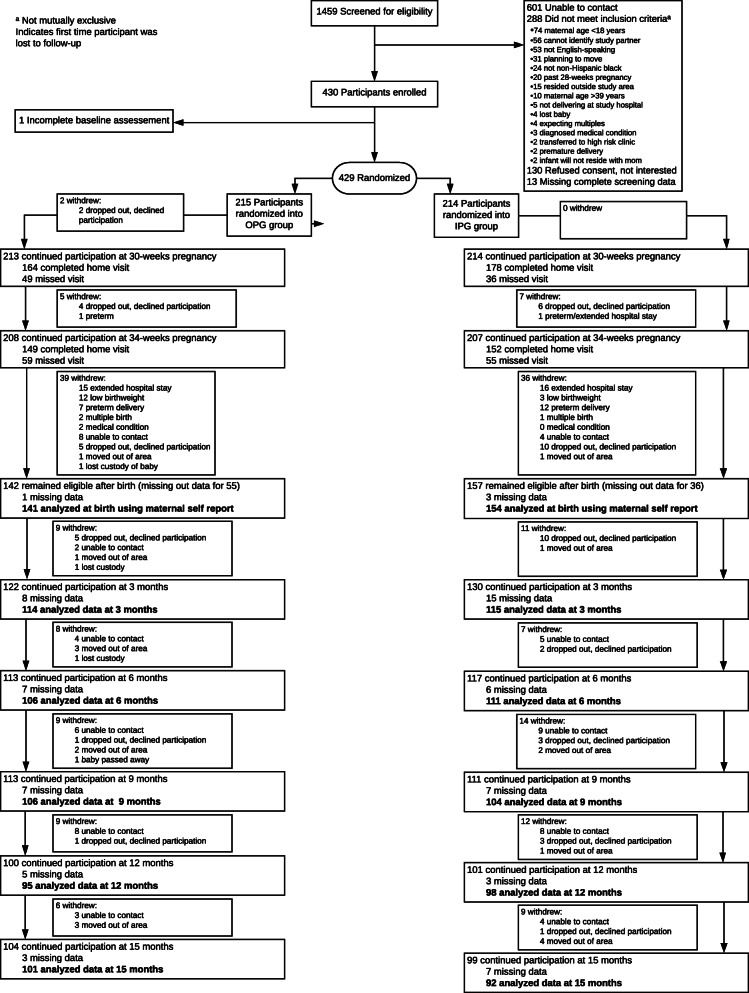
Flow diagram. ^a^Not mutually exclusive. ^b^Indicates first time that participant was lost to follow-up

Approximately 1575 women were screened in prenatal clinics, of which 1031 were eligible, although the majority (*n* = 601) could not be reached to arrange a baseline visit (Fig. 1). Four hundred and thirty women provided consent and were enrolled. One woman did not complete the baseline assessment and was not randomized and one woman was missing baseline sociodemographic data. There were no significant differences between groups in sample characteristics at baseline or in birthweight or Caesarian delivery (Table 1); however, there were differences between completers and non-completers in maternal age, pre-pregnancy body mass index (BMI), and marital status (Supplemental Table 2).

**Table 1:** Baseline and birth characteristics, overall and by treatment group

Sample characteristic	Total^**a**^ (*N* = 428)	Treatment Group	
		Obesity Prevention (*n* = 214)	Injury Prevention (*n* = 214)
*Maternal characteristics*			
Age, years, mean (SD)	25.8 (5.3)	26.2 (5.5)	25.3 (5.2)
Education, any college, No. (%)	202 (47.3)	101 (47.4)	101 (47.2)
Married, yes, No. (%)	118 (27.7)	57 (26.8)	61 (28.6)
Medicaid, yes, No. (%)	316 (74.4)	160 (74.8)	156 (73.9)
Depressive symptoms^b^, No. (%)	139 (32.8)	68 (31.9)	71 (33.7)
Currently smoke, No. (%)	37 (8.7)	21 (9.8)	16 (7.5)
Pre-pregnancy BMI, mean (SD)	28.6 (8.4)	28.4 (7.7)	28.9 (9)
First-time mother, No. (%)	239 (56.1)	116 (54.2)	123 (58)
Plan for cesarean section, No. (%)	33 (8)	18 (8.7)	15 (7.3)
*Household characteristics*			
Food insecure, No. (%)	98 (23.1)	46 (21.6)	52 (24.5)
Currently enrolled in WIC, No. (%)	349 (81.7)	179 (84)	169 (78.6)
Household size, mean (SD)	3.7 (1.7)	3.8 (1.6)	3.7 (1.7)
Dad in household, No. (%)	194 (45.2)	99 (46.1)	95 (44.4)
Grandmother in household, No. (%)	125 (29.1)	62 (28.8)	63 (29.4)
*Birth characteristics*			
Birthweight, grams, mean (SD)	3317 (430)	3306 (431)	3327 (431)
Caesarian section, No. (%)	52 (21.3)	27 (22.3)	25 (20.3)

^a^One participant did not complete the baseline assessment and was not randomized; one participant had missing sociodemographic data. ^b^Center for Epidemiological Studies-Depression scale score ≥ 16

On average, women were in their mid-twenties, had prior children, entered pregnancy with an overweight BMI, had no college education, and were single. Nearly one-third of the women reported depressive symptoms. The highest rate of withdrawal from the study was before 3-months postpartum (Fig. 1). The most common reasons were extended hospital stay (*n* = 28), refusal to continue participating (*n* = 27), loss to follow-up (*n* = 21), and preterm delivery (*n* = 20).

**Fig. 2:**
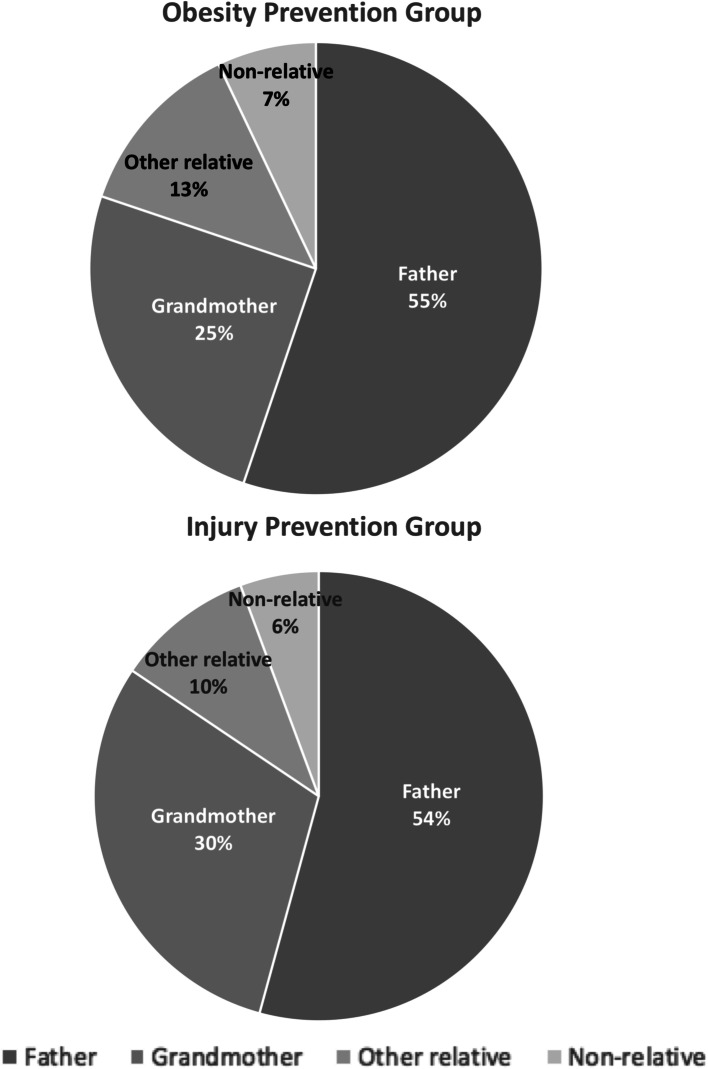
Type of study partner, by treatment group

At baseline, approximately half of women chose the infant’s father (54.6%) as their study partner, 27.5% chose the infant’s grandmother, 11.5% chose another type of relative, and 6.4% chose a nonrelative. Among the 49 women who chose an ‘other relative,’ 29 chose the infant’s aunt, 3 chose the infant’s cousin, 3 chose the infant’s grandfather, 1 chose the infant’s sister, and 13 declined to specify the type of ‘other relative.’ Of the 27 women who chose a nonrelative, 5, 2, 1, and 19 chose a roommate, the infant’s godmother, a female partner, or declined to specify the type of nonrelative, respectively. Women’s choice of study partner was similar in both treatment groups (Fig. 2).

**Fig. 3:**
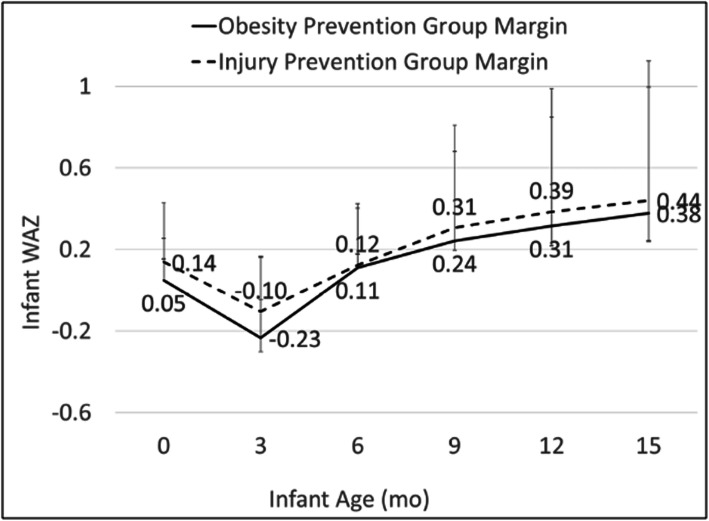
Results of adjusted mixed effects model^a,b^ examining WAZ by time and treatment. ^a^Model was adjusted for variables found to be significantly associated with the completion of any home visit and included maternal age, pre-pregnancy BMI, and marital status. The model also included breastfeeding status as a time-varying covariate. ^b^95% confidence intervals: Birth [OPG (− 0.11, 0.21), IPG (− 0.01, 0.29)], 3 months [OPG (− 0.40, − 0.07), IPG (− 0.27, 0.06)], 6 months [OPG (− 0.07, 0.29), IPG (− 0.05, 0.30)], 9 months [OPG (0.05, 0.44), IPG (0.11, 0.50)], 12 months [OPG (0.10, 0.53), IPG (0.17, 0.60)], and 15 months [OPG (0.14, 0.62), IPG (0.20, 0.68)]

At each time point, OPG infants had smaller mean WAZ scores than IPG infants, but none of the differences were statistically significant in either unadjusted or adjusted models (Table 2). A similar pattern for WAZ was seen in longitudinal models, with OPG infants demonstrating smaller, but not significant, mean WAZ scores than IPG infants (Fig. 3). Conversely, the proportion of infants categorized as overweight (WAZ ≥ 2 SD WHO) was higher, but not significantly so, at each time point (Table 3). There were also no significant differences between groups in models examining the effect of the intervention according to categorical size (lower, middle, or upper WAZ) at birth or by type of study partner (data not shown).

**Table 2:** Distribution of anthropometrics by treatment group and infant age

	Distribution of the Outcomes				Effects of the Intervention		Effects of the Intervention Accounting for Covariates^a^	
	Obesity Prevention Group		Injury Prevention Group					
	Mean (SD)	Median (IQR)	Mean (SD)	Median (IQR)	β (95% CI)	P	β (95% CI)	P
*WAZ*^*b*^								
Birth	0.00 (0.91)	−0.06 (1.42)	0.05 (0.89)	0.03 (1.36)	−.05 (−.25, .16)	.66	−.05 (−.27, .16)	.64
3 months	−0.22 (1.03)	−0.28 (1.45)	− 0.08 (0.89)	−0.12 (1.21)	−.14 (−.39, .11)	.28	−.09 (−.37, .20)	.56
6 months	0.05 (0.93)	0.07 (1.27)	0.13 (1.06)	0.22 (1.25)	−.07 (−.34, .19)	.59	−.11 (−.39, .17)	.45
9 months	0.28 (1.05)	0.30 (1.33)	0.37 (0.98)	0.44 (1.21)	−.10 (−.37, .18)	.50	−.00 (−.32, .32)	.99
12 months	0.31 (1.02)	0.19 (1.26)	0.44 (1.00)	0.44 (1.11)	−.13 (−.42, .15)	.36	−.07 (−.40, .25)	.65
15 months	0.39 (1.04)	0.20 (1.34)	0.53 (1.07)	0.56 (1.15)	−.14 (−.44, .16)	.35	−.10 (−.45, .24)	.55
*WLZ*^c^								
Birth	−1.10 (1.80)	−1.03 (2.41)	− 1.07 (1.67)	− 1.01 (2.01)	−.04 (−.45, .38)	.86	−.94 (−.52, .33)	.66
3 months	0.43 (1.33)	0.21 (1.07)	0.40 (1.63)	0.28 (1.45)	.22 (−.09, .53)	.17	.19 (−.15, .54)	.27
6 months	0.58 (1.13)	0.35 (1.22)	0.51 (1.64)	0.41 (1.20)	.23 (−.08, .55)	.15	.22 (−.11, .56)	.19
9 months	0.68 (1.09)	0.55 (1.02)	0.58 (1.51)	0.56 (1.27)	.12 (−.16, .41)	.40	.23 (−.10, .55)	.17
12 months	0.58 (1.11)	0.57 (1.07)	0.46 (1.37)	0.52 (1.19)	.01 (−.30, .32)	.96	.03 (−.32, .37)	.87
15 months	0.62 (1.11)	0.56 (1.16)	0.51 (1.22)	0.57 (1.27)	.06 (−.27, .38)	.73	.10 (−.27, .47)	.60

^a^Models were adjusted for variables found to be significantly associated with the completion of any home visit and included maternal age, pre-pregnancy BMI, and marital status. Each model after birth was also adjusted for breastfeeding, e.g. the 3-month model included a variable for continued breastfeeding at 3 months, the 6-month model included a variable for continued breastfeeding at 6 months, etc. ^b^Weight-for-age Z-score. ^c^Weight-for-length Z-score

**Table 3:** Proportion overweight by treatment group and infant age

	Distribution of the Outcomes		Effects of the Intervention		Effects of the Intervention Accounting for Covariates^a^	
	Obesity Prevention Group	Injury Prevention Group	OR (95% CI)	P	OR (95% CI)	P
	No. (%)	No. (%)				
*Overweight by WAZ*^*b,c*^						
Birth	1 (0.7)	1 (0.7)	1.09	.95	0.59	.75
3 months	3 (2.6)	3 (2.6)	1.01	.99	1.45	.78
6 months	2 (1.9)	5 (4.5)	0.41	.29	1.10	.93
9 months	9 (8.5)	5 (4.8)	1.84	.29	3.16	.17
12 months	6 (6.3)	5 (5.1)	1.25	.72	2.16	.39
15 months	8 (7.9)	7 (7.6)	1.04	.94	1.99	.34
*Overweight by WLZ*^*c,d*^						
Birth	2 (1.4)	4 (3.1)	2.19	.37	2.16	.38
3 months	9 (7.9)	5 (4.4)	1.89	.27	4.95	.16
6 months	14 (13.2)	8 (7.2)	1.96	.15	2.88	.06
9 months	15 (14.2)	9 (8.7)	1.74	.22	4.29	.03
12 months	10 (10.5)	9 (9.2)	1.16	.75	1.51	.56
15 months	13 (12.9)	9 (9.8)	1.36	.50	1.60	.39

^a^Models were adjusted for variables found to be significantly associated with the completion of any home visit and included maternal age, pre-pregnancy BMI, and marital status. Each model after birth was also adjusted for breastfeeding, e.g. the 3-month model included a variable for continued breastfeeding at 3 months, the 6-month model included a variable for continued breastfeeding at 6 months, etc. ^b^Weight-for-age Z-score. ^c^WAZ or WLZ ≥2 SD World Health Organization 2006 international growth standards [40]. ^d^Weight-for-length Z-score

**Fig. 4:**
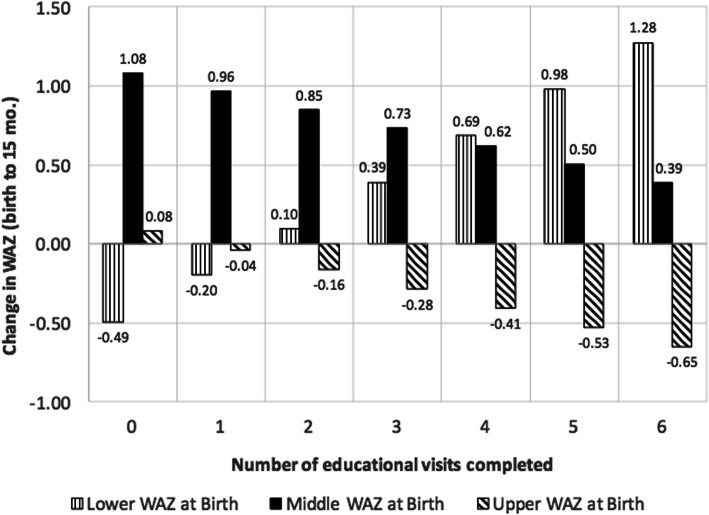
Results of adjusted linear regression model^a^ examining change in WAZ between birth and 15 months by initial size category at birth and intervention dose. ^a^Model was adjusted for variables found to be significantly associated with the completion of any home visit and included maternal age, pre-pregnancy BMI, and marital status. The model was also adjusted for breastfeeding duration

In non-ITT models (Fig. 4), infants who were in the upper end of the WAZ distribution at birth and whose mothers completed one or more visits, experienced reductions in WAZ between birth and 15 months. The size of the reduction in WAZ was greater for each additional visit completed. A similar, inverse pattern was seen for infants in the lower range of the WAZ distribution at birth. Infants in the middle range of the WAZ distribution at birth demonstrated positive mean increases in WAZ, but the increase was smaller for each additional educational home visit completed. However, the overall test of the interaction between WAZ category at birth and number of visits completed was not statistically significant (*F*_2,170_ = 1.41, *p* = 0.25).

## Discussion

Mothers & Others was an efficacy-trial of a home-based intervention designed to prevent obesity in the first year of life. Enrollment was limited to NHB, pregnant women, the majority of which were classified as low-income and single. Women chose a variety of study partners, most commonly the father and grandmother, but also aunts, cousins, grandfathers, siblings, roommates, and female partners. Although infants in the intervention group demonstrated lower mean WAZ at all assessment time points, the differences were small and did not reach statistical significance.

Our results add to similar findings from RCTs [45–47] targeting population groups at higher risk of obesity. While income was not an inclusion criterion for Mothers & Others, approximately 75% of our participants were receiving Medicaid. Two prior studies [45, 46] conducted among women participating in the Special Supplemental Nutrition Program for Women, Infants, and Children (WIC), a federal nutrition assistance program for pregnant and postpartum women, infants, and children under the age of five whose household income is ≤185% of the poverty level, documented similar null findings. Kavanagh et al. [45] targeted exclusively formula-fed infants by providing mothers a single group session covering satiety cues and instruction to feed infants under the age of 4 months less than six fluid ounces per feeding. Mothers were also given instruction on non-food techniques for soothing a crying baby [48]. At approximately 4-months-old, infants in their intervention group had gained significantly more weight than infants in their control group (195.3 ± 10.0 g vs 156.1 ± 9.5 g, respectively, *p* = .008).

In a study similar in design to Mothers & Others, Reifsnider et al. (2018) [49] targeted infants of obese, Hispanic, pregnant women enrolled in WIC. The intervention consisted of eight home visits delivered by peer educators, or “promotorás,” who charted infant weight and length and provided advice on infant feeding, play, and sleep. Mothers could also request the services of a LC. At 12 months of age, there were no differences between infants in the intervention versus control group in WLZ or proportion of infants classified as overweight or obese [46].

In the Australian study, Healthy Beginnings, Wen et al. [50] targeted all pregnant women attending antenatal clinics serving a disadvantaged population. The intervention group received eight postpartum home visits from a community health nurse. At 2 years of age, there was a small, statistically significant difference in BMI between children in the intervention and control group (BMI_diff_ = − 0.29, 95% CI: − 0.55, − 0.02) [47]. An important difference between Healthy Beginnings and Mothers & Others is the proportion of women who were married: 90% versus 28%, respectively. Single mothers often report higher levels of stress than do married mothers [51], with higher maternal stress associated with more controlling feeding practices [52] and child weight status [53, 54], particularly among NHB children and children from low-income families [55].

Results from RCTs [56–61] conducted among more economically advantaged populations have been mixed. Two trials [62, 63] in Australia, each utilizing first-time parent groups to deliver AG on infant feeding and physical activity between approximately 4–18 months postpartum, produced null findings [56, 57, 60]. Conversely, two trials, POI in New Zealand [64] and INSIGHT in the U.S. [65], both of which delivered the intervention through home visits from research nurses and had a strong component on infant sleep, documented positive outcomes for infant size and growth [58, 59, 61]. Of note, the majority of participants in each of these studies had attended college (76% in POI and 90% in INISGHT), and 75% of the mothers in INSIGHT were married. Thus, home visits by research nurses appear a promising strategy among more economically advantaged or married, NHW populations. It is not known if such interventions are effective among lower-income or racially diverse populations.

A novel component of Mothers & Others was the active inclusion of a study partner in the intervention group; however, our results suggest support provided by the PEs was beneficial in both arms. Prior studies [66, 67] of organized peer support, particularly among low-income and minority women, have shown it to reduce social isolation, provide validation of parenting practices, and enhance the emotional well-being of mothers. It is possible that organized peer support, regardless of the topic, may be beneficial for obesity prevention through its role in enhancing maternal emotional well-being. Research appropriately designed to test this hypothesis is needed.

It is also important to note the multicomponent design of the trials published to date as well as the seemingly different developmental philosophies occurring across, and within, trials. The Multiphase Optimization Strategy, or MOST, is a framework for optimizing the design of behavioral interventions [68]. Inspired by engineering, MOST encourages designs, often a factorial experiment, which can isolate the effect of individual intervention components. In Mothers & Others, a factorial experiment could have yielded individual main effects for components, and interactions between components, such as those designed to promote responsive feeding versus those targeting maternal social support. It is possible some content in Mothers & Others had no effect or was even antagonistic; this is important information for moving the science forward.

For future research, we also believe it important to test the seemingly different developmental approaches trials have used to address infant fussing and crying. For example, one could test the difference in helping parents understand what their infant is communicating, thereby promoting the developmental philosophy of mind-mindedness [69], versus provision of strategies to minimize crying [48]. Meins et al. (2001) define mind-mindedness as “the mother’s proclivity to treat her infant as an individual with a mind, rather than merely as a creature with needs that must be satisfied” (p. 638) [70]. A substantial body of research has demonstrated positive associations between parental mind-mindedness and child attachment [71–73], emotion regulation [74], and executive functioning [75, 76], as well as maternal responsive feeding behaviors [77]. Given its import to multiple domains of child development, it seems judicious to test the extent to which different intervention approaches impact both mind-mindedness and weight status.

Mothers & Others contained several limitations. First, we did not report WLZ, given the systematic differences in length between the two arms. Because the PEs each collected anthropometric measurements of infants in their respective arms, we cannot be certain whether the differences in length were due to biological differences or differences in measurement. Having the PEs collect the anthropometric data also raises a concern for bias. However, several lines of reasoning and examination point to differences in measurement technique rather than bias. The length of time between visits (3 months) makes it unlikely that PEs remembered infant weight or length values at prior visits. Also, the fact that the error was in length rather than weight variables makes it unlikely that bias, rather than differences in technique, contributed to the systematic error. Further, the intra-rater reliability was very high for both PEs and remained high from the initial to final visits, suggesting that drift in technique was also not the cause of the differences between groups. All other measures were completed prior to the home visit via online (majority) or mailed surveys. Second, birthweight data was planned to be collected from the medical record by trained research staff in our research network. This staff was also responsible for screening and recruitment, which took longer than anticipated, thus exhausting funds for medical record extraction. Third, generalizability of our findings is limited to predominately low-income, NHB women; however, this is an important population for early life obesity prevention. Finally, attrition was high in Mothers & Others, although not differential across treatment arms, and may have limited power to detect differences in infant size and growth. In their study of low-income, Hispanic mother-infant dyads, Reifsnider et al. [46] reported a similar rate of retention (64%) at 12 months. Together, these trials provide important data on recruitment and retention for future studies targeting similar income and racial/ethnic populations. Examination of secondary outcomes, including maternal and study partner behaviors related to infant feeding, movement, and screen exposure are underway, as are analyses of mediators (e.g. change in knowledge, infant feeding styles, and infant feeding attitudes). Data on injury prevention practices were also collected and may be examined to determine the efficacy of the attention-control group in this regard.

## Conclusions

Mothers & Others did not produce significant differences in infant size or growth during the first 15 months of life, a result consistent with other trials conducted among low-income, minority populations in the US, of which there have been few. Although we recruited a large sample and had high retention during the postpartum period, there was high attrition between the last prenatal and first postpartum home visit. Additional home visits by the PE, a person with which the mothers had built rapport, earlier in the postpartum period may be a critical component for future studies. Indeed, our finding in both groups of positive, incremental effects on infant size by number of PE home visits received suggests an important role of organized peer support, regardless of the content provided. Finally, that women chose a variety of study partners, including but not limited to fathers and grandmothers, suggests a need for interventions and observational research that recognize the varied circumstances of women.

## Supplementary information

**Additional file 1: Supplemental Table 1.** Intervention content, by timing of delivery and study arm.

**Additional file 2: Table S2.** Sample characteristics of eligible participants^a^ by completion of home visits.

## Data Availability

The dataset used and analyzed during the current study is available from the corresponding author on reasonable request.

## Abbreviations

AG: Anticipatory guidance
BF: Breastfeeding
BMI: Body mass index
CF: Complementary foods
EBF: Exclusive breastfeeding
INSIGHT: Intervention Nurses Start Infants Growing on Healthy Trajectories
IPG: Injury prevention group
ITT: Intent-to-treat
L&D: Labor and delivery
LAZ: Length-for-age z-score
LC: Lactation consultant
MOST: Multiphase Optimization Strategy
NHANES: National Health and Nutrition Examination Survey
NHB: Non-Hispanic black
NHW: Non-Hispanic white
OPG: Obesity prevention group
PE: Peer educator
POI: Prevention of Overweight in Infancy
RCT: Randomized controlled trial
WAZ: Weight-for-age z-score
WLZ: Weight-for-length z-score
WIC: Special Supplemental Nutrition Program for Women, Infants, and Children
