# Supplementary material for: Home-based intervention for non-Hispanic black families finds no significant difference in infant size or growth: results from the Mothers & Others randomized controlled trial
Source: BMC Pediatr. 2020 Aug 18;20:385. doi: 10.1186/s12887-020-02273-9 (PMC7433206; doi:10.1186/s12887-020-02273-9)
Supplement: Supplementary file 1 — Additional file 1: Supplemental Table 1. Intervention content, by timing of delivery and study arm. [file 12887_2020_2273_MOESM1_ESM.docx]

Supplemental Table 1. Intervention content, by timing of delivery and study arm.

| **Type of contact** | **Obesity Prevention Group**  **(Intervention Arm)** | **Injury Prevention Group**  **(Attention-Control Arm)** |
| --- | --- | --- |
| **30 week prenatal home visit** | - Baby Behavior: sleep, crying and cues – including responsive feeding - Importance of play and minimizing screen time - Mobilizing social support - Introducing you to your toolkit: finding more information | - Preventing SIDS and accidental suffocation - Tips for selecting a crib - Introducing you to your toolkit: finding more information |
| **34 week prenatal home visit** | - Benefits of breastfeeding/exclusive breastfeeding and what to expect in the first few days and weeks - Informed decision-making - Reinforce responsive feeding | - Choosing and using a safe car seat - Safety in and around the car - Keeping baby safe in a stroller |
| **2 month newsletter** | - Complementary feeding overview for first year of life, delay solids until 6 months - Ages & Stages developmental activities for 2-4 month-olds - Developmental milestones and when to be concerned | - Safety overview for first year of life - Developmental milestones and when to be concerned |
| **3 month home visit** | - Review 2-month newsletter - Healthy family behaviors (maternal diet) | - Top safety tips for 1–6 months: the “head up” stage - Getting ahead on safety: childproofing your home for the “independent sitter” stage |
| **5 month newsletter** | - Complementary feeding (supported sitter stage): iron-rich foods, keep breastfeeding, feeding cues for older babies, mealtime learning, limit juice - ASQ activities for 4-8 month-olds - Developmental milestones and when to be concerned | - Developmental milestones and when to be concerned |
| **6 month home visit** | - Review 5-month newsletter - Healthy family behaviors (maternal physical activity, limiting screen time; check-in on maternal diet) - Baby Behavior: Cues for older babies, learning through movement, social referencing, reasons for crying and how to soothe, common sleep patterns and tips for getting back to bed | - Let's review: top safety tips for the “independent sitter” stage - Getting ahead on safety: childproofing your home for the “crawler” stage - Home fire safety: prepare, practice, prevent the unthinkable |
| **7 month newsletter** | - Complementary feeding (crawler stage): finger foods, vegetables for family and baby, establishing mealtime routines, healthy bottle and sippy cup behaviors - Ages & Stages developmental activities for 8-12 month-olds - Developmental milestones and when to be concerned | - Developmental milestones and when to be concerned |
| **9 month home visit** | - Review 7-month newsletter - Healthy family behaviors (check-in on maternal diet, physical activity, limiting screen time) - Baby Behavior: Reasons for not eating/“picky” eating, importance of routines (not schedules) | - Let's review: top safety tips for the “crawler” stage - Getting ahead on safety: childproofing your home for the “learning to walk” stage - Preventing TV tip-overs: what every parent should know |
| **10 month newsletter** | - Complementary feeding (beginning to walk): sample menus, responsive feeding and avoiding food struggles, switching from bottle to cup, weaning - Ages & Stages developmental activities for 12-16 month-olds - Developmental milestones and when to be concerned | - Developmental milestones and when to be concerned |
| **12 month home visit** | - Review 10-month newsletter - Healthy family behaviors (check-in on maternal diet, physical activity, limiting screen time) - Eating more together as a family | - Let's review: childproofing your home - Splish-splash: Staying safe around water - Bye-bye boo-boos: staying safe on the playground |
| **15 month mailing** | - Ages & Stages developmental activities for 16-20 month-olds - Developmental milestones and when to be concerned | - Developmental milestones and when to be concerned |
