# Supplementary material for: Home-based intervention for non-Hispanic black families finds no significant difference in infant size or growth: results from the Mothers & Others randomized controlled trial
Source: BMC Pediatr. 2020 Aug 18;20:385. doi: 10.1186/s12887-020-02273-9 (PMC7433206; doi:10.1186/s12887-020-02273-9)
Supplement: Supplementary file 2 — Additional file 2: Table S2. Sample characteristics of eligible participantsa by completion of home visits. [file 12887_2020_2273_MOESM2_ESM.docx]

**Table S2.** Sample characteristics of eligible participants^a^ by completion of home visits

|  | 30 weeks |  | 34 weeks |  | 3 months |  | 6 months |  | 9 months |  | 12 months |  | 15 months |  |
| --- | --- | --- | --- | --- | --- | --- | --- | --- | --- | --- | --- | --- | --- | --- |
|  | Yes | No | Yes | No | Yes | No | Yes | No | Yes | No | Yes | No | Yes | No |
| Total | 261 (87.3) | 38 (12.7) | 245 (81.9) | 54 (18.1) | 229 (76.6) | 70 (23.4) | 217 (72.6) | 82 (27.4) | 210 (70.2) | 89 (29.8) | 193 (64.6) | 106 (35.5) | 193 (64.6) | 106 (35.5) |
| *Maternal characteristics* |  |  |  |  |  |  |  |  |  |  |  |  |  |  |
| Age, years, mean (SD) | 25.8 (5.2) | 25.1 (5.0) | 25.8 (5.2) | 25.8 (5.0) | **26.1 (5.3)^d^** | **24.4 ± (4.5)** | 25.0 (5.2) | 25.0 (5.0) | 25.9 (5.1) | 25.2 (5.3) | 25.7 (5.2) | 25.6 (5.2) | 25.8 (5.2) | 25.4 (5.2) |
| Education, any college, No. (%) | 135 (51.7) | 16 (42.1) | 129 (52.7) | 22 (40.7) | 123 (53.7) | 28 (40.0) | 114 (52.5) | 37 (45.1) | 104 (49.5) | 47 (52.8) | 96 (49.7) | 55 (51.9) | 98 (50.8) | 53 (50.0) |
| Married, yes, No. (%) | 82 (31.5) | 8 (21.1) | 78 (32.0) | 12 (22.2) | **77 (33.8)^d^** | **13 (18.6)** | 66 (30.6) | 24 (29.3) | 66 (31.6) | 24 (27.0) | 57 (29.5) | 33 (31.4) | 59 (30.6) | 31 (29.5) |
| Medicaid, yes, No. (%) | 187 (72.5) | 26 (68.4) | 171 (70.7) | 42 (77.8) | 164 (72.3) | 49 (71.0) | 151 (70.2) | 62 (76.5) | 147 (70.7) | 66 (75.0) | 132 (69.1) | 81 (77.1) | 132 (69.1) | 81 (77.1) |
| Depressive symptoms, No. (%) | 70 (27.2) | 10 (26.3) | 65 (26.9) | 15 (28.3) | 61 (27.0) | 19 (27.5) | 59 (27.6) | 21 (25.9) | 54 (26.1) | 26 (29.6) | 50 (26.2) | 30 (28.9) | 50 (26.2) | 30 (28.9) |
| Currently smoke, No. (%) | 18 (6.9) | 5 (13.2) | 17 (7.0) | 6 (11.1) | 17 (7.5) | 6 (8.6) | 16 (7.4) | 7 (7) | 16 (7.7) | 7 (7.9) | 14 (7.3) | 9 (8.5) | 13 (6.8) | 10 (9.4) |
| Pre-pregnancy BMI, mean (SD) | **29.1 (7.9)^d^** | **26.2 (7.5)** | 29.1 (7.9) | 26.9 (7.6) | **29.4 (7.9)^c^** | **26.3 (7.4)** | **29.7 (8.0)^b^** | **26.0 (6.9)** | **29.6 (8.3)^c^** | **26.7 (6.6)** | **29.4 (8.1)^d^** | **27.4 (7.4)** | **29.6 (8.3)^d^** | **27.1 (6.9)** |
| First-time mother, No. (%) | 142 (54.8) | 22 (57.9) | 132 (54.3) | 32 (59.3) | 127 (55.7) | 37 (53.6) | 122 (56.5) | 42 (51.9) | 117 (56.0) | 47 (53.4) | 103 (53.7) | 61 (58.1) | 103 (53.7) | 61 (58.1) |
| Plan for cesarean section, No. (%) | 19 (7.5) | 2 (5.7) | 19 (8.0) | 2 (4.0) | 18 (8.0) | 3 (4.6) | 16 (7.6) | 5 (6.3) | 14 (6.9) | 7 (8.1) | 13 (7.0) | 8 (7.8) | 13 (7.0) | 8 (7.8) |
| *Household characteristics* |  |  |  |  |  |  |  |  |  |  |  |  |  |  |
| Food insecure, No. (%) | 57 (21.9) | 9 (24.3) | 55 (22.5) | 11 (20.8) | 45 (19.7) | 21 (30.4) | 47 (21.8) | 19 (23.5) | 42 (20.1) | 24 (27.3) | 36 (18.8) | 30 (28.6) | 39 (20.3) | 27 (25.7) |
| Household size, mean (SD) | 3.8 (1.7) | 3.6 (1.6) | 3.8 (1.7) | 3.7 (1.7) | 3.7 (1.7) | 3.7 (1.6) | 3.7 (1.7) | 3.8 (1.6) | 3.8 (1.7) | 3.6 (1.6) | 3.7 (1.7) | 3.7 (1.7) | 3.7 (1.7) | 3.8 (1.7) |
| Dad in household, No. (%) | 124 (47.5) | 17 (44.7) | 118 (48.2) | 23 (42.6) | 113 (49.3) | 28 (40.0) | 100 (46.1) | 41 (50.0) | 103 (49.1) | 38 (42.7) | 88 (45.6) | 53 (50.0) | 93 (48.2) | 48 (45.3) |
| Grandmother in household, No. (%) | 81 (31.0) | 7 (18.4) | 75 (30.6) | 13 (24.1) | 65 (28.4) | 23 (32.9) | 65 (30.0) | 23 (28.1) | 64 (30.5) | 24 (27.0) | 61 (31.6) | 27 (25.5) | 59 (30.6) | 29 (27.4) |

^a^ Data is for 299 participants who remained eligible after application of labor and delivery criteria

^b^ *P*<.001

^c^ *P*<.01

^d^ *P*<.05
